# Choroidal neovascularization in a patient after resolution of multiple evanescent white dot syndrome: A case report

**DOI:** 10.1002/ccr3.5802

**Published:** 2022-05-15

**Authors:** Marie Burova, Alexandr Stepanov, Basma Almesmary, Nada Jiraskova

**Affiliations:** ^1^ Department of Ophthalmology Faculty Hospital in Hradec Kralove and Charles University Faculty of Medicine in Hradec Kralove Hradec Kralove Czech Republic

**Keywords:** choroidal neovascularization, inflammatory, multiple evanescent white dot syndrome, ranibizumab, self‐limiting

## Abstract

Choroidal neovascularization (CNV) is a rare complication of the Multiple Evanescent White Dot Syndrome (MEWDS). It can develop after resolving of the disease when there is already no evident inflammatory activity. Therefore, a long‐term follow‐up of such patients is important.

## Graphical abstract

Choroidal neovascularization (CNV) is a rare complication of the multiple evanescent white dot syndrome (MEWDS). It can develop even when there is no evident inflammatory activity. Therefore, a long‐term follow‐up of such patients is important.

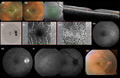

## 1 INTRODUCTION

Multiple evanescent white dot syndrome is a rare inflammatory retinal disease first described by Jampol et al. in 1984.^1^ It is characterized by multifocal gray‐white dots (measuring 100 to 200 μm) at the level of retinal pigment epithelium or deep retina with typical RPE granularity in the fovea. Mild anterior chamber flare with mild vitritis and optic disc edema may also be seen. In rare cases, sheathing of retinal veins is present. The disease is usually unilateral and affects young patients, predominantly myopic women. Its main symptoms are acute painless decrease of vision, photopsias, temporal or paracentral scotoma, enlarged blind spot, and dyschromatopsia.^1^, ^3^, ^15^, ^16^

Although the condition is self‐limiting and has good visual prognosis, a complication such as choroidal neovascular membrane can develop.^10^, ^12^, ^13^, ^15^, ^16^

## 2 CASE DESCRIPTION

**FIGURE 1:**
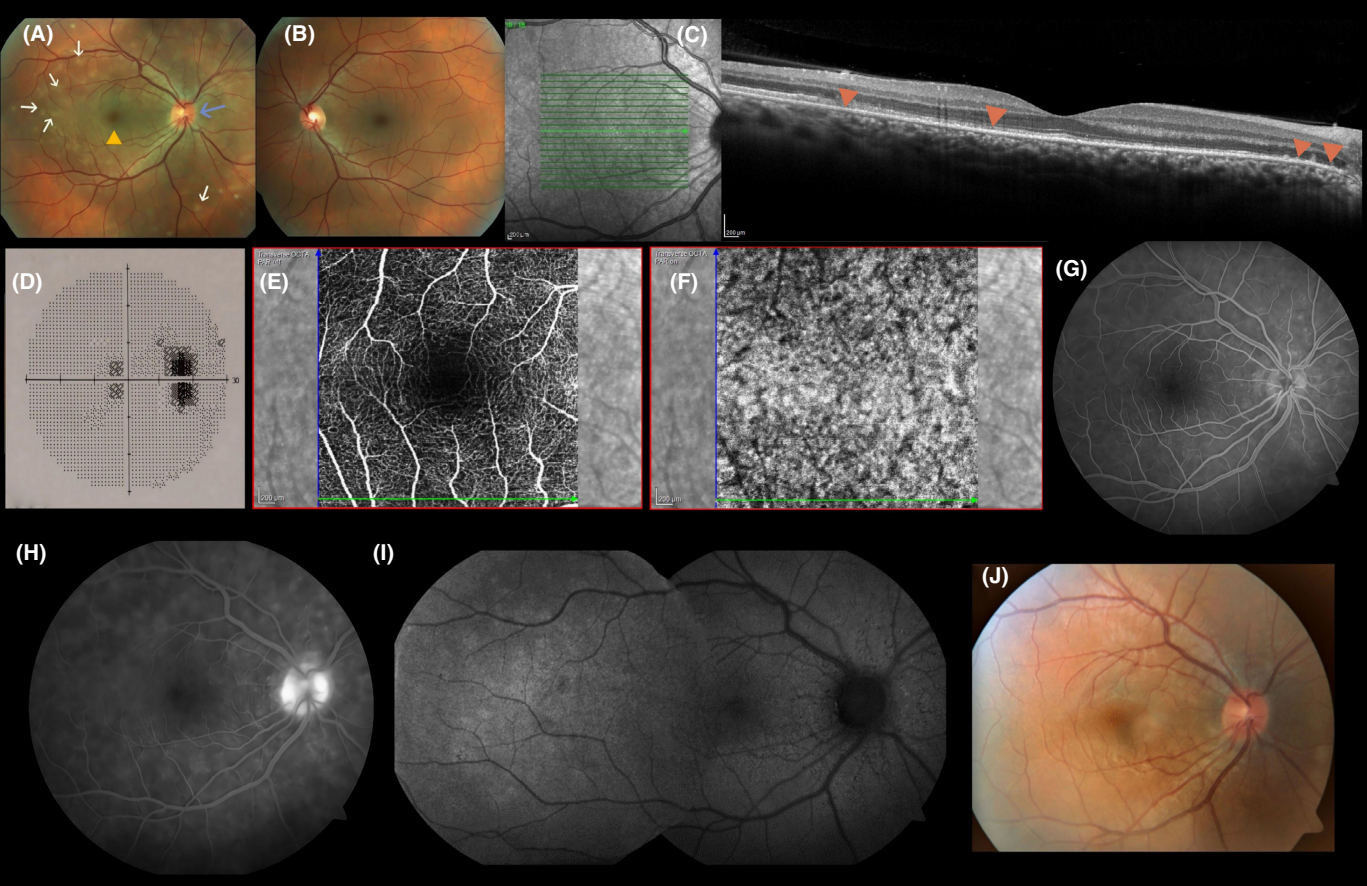
(A). Fundus photography of the right eye shows multiple gray‐white spots in the posterior pole (white arrows), foveal granularity (yellow arrowhead), and slight optic disc edema (blue arrow). (B) Fundus photography of the left eye is normal. (C) OCT demonstrates irregular photoreceptor ellipsoid zone disruptions in subfoveal and peripapillary area (red arrowheads). (D) Visual field test of the right eye demonstrates an enlargement of the blind spot and central scotoma. (E,F) OCTA shows normal retinal and choroidal vasculature. (G) An early‐phase FA image shows punctate hyperfluorescence. (H) A late‐phase FA image shows staining of lesions and optic disc leakage. (I) Fundus autofluorescence of the right eye. (J) Fundus photography of the right eye shows resolving of gray‐white lesions

A 26‐year‐old previously healthy man presented to the ophthalmology clinic of the Faculty Hospital in Hradec Kralove (Czech Republic) with a 1‐week history of worsening vision, metamorphopsias, and dyschromatopsia in the right eye. He denied prodromal symptoms or vaccination. The past medical, drug, and family histories were unremarkable. The patient had myopia with refractive correction −0.75D in both eyes. His best‐corrected visual acuity (BCVA) was 20/20 in both eyes. Anterior segment including pupillary reaction was normal on both eyes. A fundus examination of the right eye revealed mild vitreous cells, slight optic disc edema, foveal granularity with orange hue, and multiple gray‐white spots in the posterior pole (Figure 1A). A fundus examination of the left eye was normal (Figure 1B). Digital color fundus photography (CFP) was performed in both eyes. Optical coherence tomography (OCT) of the right eye showed irregular photoreceptor ellipsoid zone disruptions in the subfoveal and peripapillary area (Figure 1C), which were corresponded to blind spot enlargement and central scotoma in the visual field test (Figure 1D). OCT of the left eye did not shows any pathology. OCT angiography (OCTA) of the right eye did not reveal flow impairment in the retinal and choroidal vasculature (Figure 1E,F). Fluorescein angiography (FA) of the right eye demonstrated early punctate hyperfluorescence with late staining of lesions and optic disc leakage as well (Figure 1G,H). Fundus autofluorescence (FAF) of the right eye showed multiple hyperautofluorescent spots mainly in the temporal part of the posterior pole and small hypoautofluorescent lesions around the optic disc (Figure 1I). OCT, OCTA, FA, FAF, and visual field test of the left eye were unremarkable. After all above‐mentioned examinations, MEWDS was diagnosed. Since it is a self‐limiting disease, the patient was only observed. BCVA of the right eye improved to 20/16, all symptoms and fundus pathological findings disappeared within 1 month (Figure 1J).

**FIGURE 2:**
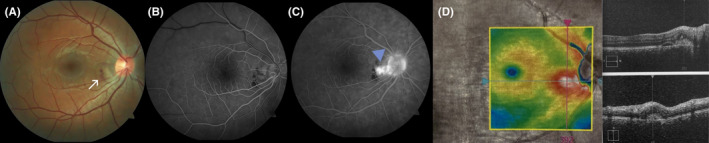
(A) Fundus photography of the right eye shows small peripapillary subretinal hemorrhage (white arrow). (B,C) An early‐ and late‐phase FA images show hyperfluorescence of classic CNV, which progressively intensifies with leakage of dye (blue arrowhead). (D) OCT demonstrates classic CNV with sub‐ and intra‐retinal fluid

However, 2 months after the first visit of patient a small peripapillary subretinal hemorrhage on the fundus examination of the right eye was noted (Figure 2A). OCT and FA confirmed the presence of the classic peripapillary CNV (Figure 2B–D). Because of good BCVA of the right eye (20/16), absence of any complaints, and peripapillary localization of the CNV, we proceeded in observation with 1‐month follow‐up interval. Though the treatment of CNV in patients with normal vision is also an option.

**FIGURE 3:**
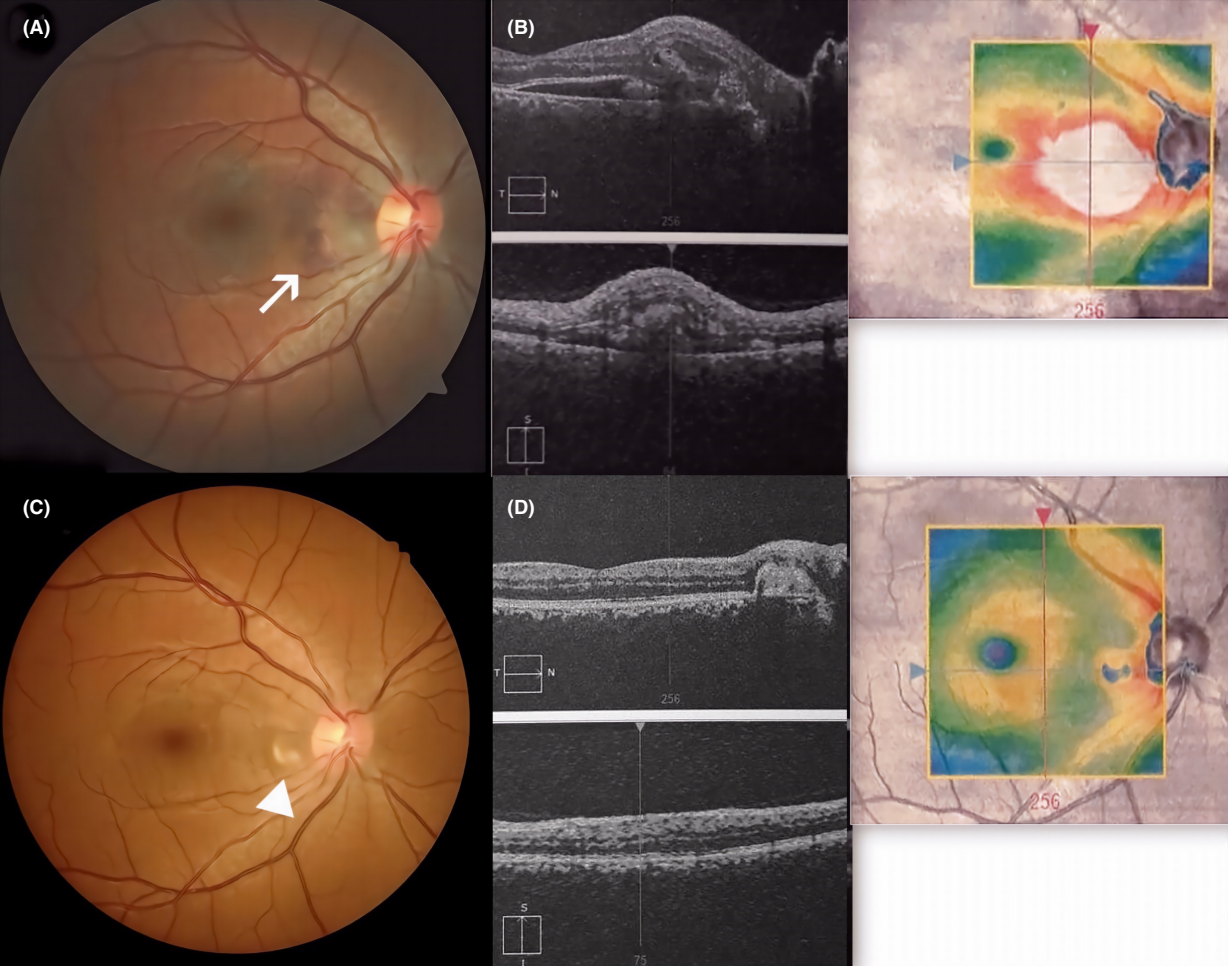
(A) Fundus photography shows extension of peripapillary subretinal hemorrhage (white arrow). (B) OCT shows more extensive subretinal and intraretinal fluid accumulation. (C,D) Fundus photography and OCT of the right eye after 4 injections of ranibizumab show fibrous scarring of inactive peripapillary CNV (white arrowhead)

Within 6 months of follow‐up, impairment of BCVA of the right eye occurred (20/40). Patient complained of significant metamorphopsias. A fundus examination of the right eye revealed larger peripapillary subretinal hemorrhage (Figure 3A). OCT showed extensive subretinal and intraretinal fluid accumulation between macula and optic disc (Figure 3B). Therefore, intravitreal anti‐VEGF treatment was indicated. Within the following 5 months, 4 ranibizumab (Lucentis, Novartis) injections (0.5 mg/0.05 ml in one dose) were administered. After the last injection BCVA of the right eye improved to 20/20, metamorphopsias disappeared. Control fundus examination and OCT demonstrated resolving of intraretinal and subretinal fluid and hemorrhage as well.

At the last visit (September 2020, 1 year after the 1^st^ injection), our patient did not have any complaints, BCVA of the right eye remained 20/20, OCT and fundus examination confirmed the presence of a small scar of inactive CNV (Figure 3C,D).

## 3 DISCUSSION AND CONCLUSION

Multiple Evanescent White Dot Syndrome is a rare condition characterized by an idiopathic inflammatory retinal disorder, which belongs to the group of White Dot Syndromes. Usually unilateral, bilateral cases, however, have been also reported.^2^ There are not any racial or regional predilections. The etiology and exact pathogenesis still remain uncertain. Although viral infection and vaccination are supposed to be possible trigger factors.^3^, ^4^, ^12^ Several cases of MEWDS following vaccination against influenza, meningococcus, yellow fever, human papillomavirus, rabies, and hepatitis A and B have been described in the literature.^4^ Regarding the pathogenesis, it is considered that MEWDS affects the RPE and outer retina. Lages et al. supposed that choriocapillaris hypoperfusion or non‐perfusion takes place, which results in ischemic damage to the outer retina and RPE due to a vaso‐occlusive process in small vessels.^5^ Hashimoto et al. suggested a probable role of inflammation‐related choroidal circulation impairment in the outer retinal disorder in MEWDS. This author also described the choroidal thickness increasing during the acute phase of the disease.^6^, ^7^

Multiple Evanescent White Dot Syndrome should be differentiated from other white dot syndromes such as multifocal choroiditis and panuveitis (MCP), punctate inner choroiditis (PIC), acute multifocal placoid pigment epitheliopathy (AMPPE), serpiginous choroiditis (SC), birdshot chorioretinopathy (BC), and acute zonal occult outer retinopathy (AZOOR).^8^MEWDS is a self‐limiting disease with an excellent visual prognosis, but a rare complication as CNV can occur, commonly type 2 membrane. The proposed mechanism of development of CNV in such cases is the focal disruption in the Bruch's membrane and RPE due to choroidal inflammation stimulating new vessels growth. The severity of visual impairment caused by CNV depends on its localization. OCT, OCT angiography, FA, Indocyanine green angiography (ICGA), and FAF are undoubtedly useful both in diagnostics of MEWDS and in detection/evaluation of CNV. Intravitreal application of VEGF‐inhibitors is a highly efficacious treatment option for the management of CNV associated with white dot syndromes (in our case with MEWDS).^9^, ^10^, ^11^ Rouvas et al. described a complete regression of CNV secondary to MEWDS after a single injection of ranibizumab.^12^ Parodi et al. also presented good clinical outcomes of intravitreal ranibizumab treatment in 4 patients with MEWDS complicated by CNV.^13^ Mansour et al. in their case series demonstrated significant visual improvement and regression inflammatory CNV after intravitreal application of bevacizumab (on average 1–3 injections).^14^ However, due to its inflammatory origin the main principle of the treatment is to suppress the inflammation with steroids (systemic and local if the process is unilateral) and/or immunosupressants.^9^, ^10^ In a case reported by Savastano et al., juxtapapillary CNV in MEWDS was significantly regressed after administering of prednisolone orally.^15^ Papadia et al. in their case used a combination of intravitreal bevacizumab (Avastin, Genentech) and systemic corticosteroid (Prednisone).^16^ Performing of photodynamic therapy (PDT) nowadays is uncommon for its possible adverse effects (choriocapillaris hypoperfusion, atrophy of retinal pigment epithelium, and increased subretinal edema followed by foveal thinning).^9^, ^10^, ^11^

In case, we presented a peripapillary classic CNV after apparent resolving of MEWDS suddenly developed. There were no chorioretinal scars or atrophy at this region. It is known that growth of new blood vessels can occur even when there is no evident inflammatory activity.^10^ For diagnostics and evaluation of CNV dynamics, CFP, OCT, and FA were made. In our case, we did not perform ICGA and we did not notice any pathological choroidal findings on OCTA. Nevertheless, we believe that the subclinical choroidal inflammation was present and could cause both hypoperfusion of choriocapillaris and disruption in the Bruch's membrane. As a result, there was increased vascular endothelial growth factors production stimulating the neoangiogenic process.^9^, ^10^ The decision to use anti‐VEGF agents without using corticosteroids or immunosuppressants was made. After 4 injections of ranibizumab administered within 5 months, complete regression of the CNV was observed.

In conclusion, we reported a rare case of MEWDS complicated by growth of the peripapillary CNV. It is necessary to keep in mind that the process of choroidal neoangiogenesis in uveitis can take place even when there is no apparently evident inflammation. Therefore, a long‐term follow‐up of such patients is important. Anti‐VEGF‐therapy seems highly effective in the management of CNV associated with MEWDS.

## CONFLICT OF INTEREST

The authors report no conflicts of interest. The authors alone are responsible for the content and writing of the paper.

## AUTHOR CONTRIBUTIONS

MB and AS performed examination of the patient and wrote the paper. BA wrote the paper. NJ revised the paper and approved the final version to be published.

## CONSENT

Written informed consent was obtained from the patient to publish this report in accordance with the journal's patient consent policy.
